# The host ubiquitination system dynamically regulates the inflammatory response of macrophages to bacteria

**DOI:** 10.3389/fimmu.2026.1831712

**Published:** 2026-05-11

**Authors:** Jinru Liu, Xiaomei Li, Luna He, Xin Wang, Hongyuan Yang, Xianggui Yang, Jun Zeng

**Affiliations:** 1 Division of Pulmonary and Critical Care Medicine, Clinical Medical College and The First Affiliated Hospital of Chengdu Medical College, Chengdu, China; 2 School of Clinical Medicine, Chengdu Medical College, Chengdu, China; 3 Department of Laboratory Medicine, Clinical Medical College and The First Affiliated Hospital of Chengdu Medical College, Chengdu, China

**Keywords:** ubiquitination system, macrophages, TLR/NF-κB pathway, xenophagy, E3 ubiquitin ligase, deubiquitinating enzyme

## Abstract

Resistant bacterial infections have become a major global public health challenge, claiming millions of lives annually and imposing enormous economic burdens. The core issue lies in the imbalance of the host immune system, particularly macrophage function. This review elucidates the pivotal role of the host ubiquitin system in macrophage antimicrobial immunity. It systematically examines how this system leverages its unique enzyme-substrate network to orchestrate immune responses with dynamic equilibrium and precision, achieved through two critical pathways: the appropriate modulation of inflammatory signaling and the targeted clearance of intracellular pathogens. Within the Toll-like receptor (TLR)/Nuclear factor kappa-B (NF-κB) pathway, the ubiquitination system initiates inflammatory responses by activating molecules such as TRAF6, while undergoing negative feedback regulation via deubiquitinating enzymes like A20 to prevent excessive inflammatory damage. Within the autophagy pathway, ubiquitination functions as a “targeting system,” where ubiquitin ligases like Parkin and RNF213 mark and eliminate intracellular bacteria such as (*Mtb*) and *Salmonella*. In-depth analysis of the ubiquitin system’s specific roles in infection immunity and distinct bacterial infections holds significant importance for elucidating the molecular mechanisms underlying host-pathogen interactions. It will also provide key targets and novel perspectives for developing novel therapeutic strategies against drug-resistant bacterial infections.

## 1 Introduction

Resistant bacterial infections have become a major global public health challenge. According to the World Health Organization (WHO), millions of lives are lost annually due to these infections, which also impose a significant economic burden (1). An imbalance in the host immune system—particularly dysfunction of macrophages—can significantly exacerbate the severity of infection, compromise pathogen clearance, and contribute to adverse clinical outcomes (2). In bacterial infections, the host E3 ubiquitin ligase Hrd1 plays a critical role in host immune defense by promoting TLR-induced inflammatory responses (3); the E3 ubiquitin ligase Cbl-b negatively regulates TLR signaling through ubiquitination, and its dysfunction can lead to uncontrolled inflammation (4). Furthermore, impaired TRIM21 function allows bacterial effector proteins to evade host clearance (5). These clinical observations collectively reveal a fundamental challenge: macrophages must initiate intense inflammatory and autophagic responses to clear invading bacteria, but excessive activation may lead to damage to host tissues. By dynamically regulating the intensity and duration of macrophages’ inflammatory response to bacteria, the host ubiquitination system serves as a key molecular switch for resolving this core dilemma.

To understand how this molecular switch works, it is necessary to first provide a brief overview of the ubiquitination system. Ubiquitin is a polypeptide consisting of 76 amino acids that can be covalently attached to lysine residues on target proteins—a process known as ubiquitination. This post-translational modification regulates protein degradation, signal transduction, vesicular transport, and autophagy. Ubiquitination proceeds through a three-enzyme cascade: an E1 activase (UBA1 or UBA6 in mammals) activates ubiquitin in an ATP-dependent manner and transfers it to an E2 ligase; subsequently, the E2 enzyme acts in concert with the E3 ubiquitin ligase, which recognizes specific substrates and catalyzes the final transfer of ubiquitin. The E3 ligase determines the specificity of ubiquitination and is therefore the most extensively studied component, as illustrated by Hrd1, Cbl-b, and TRIM21 mentioned above. Ubiquitin itself contains seven lysine residues (K6, K11, K27, K29, K33, K48, K63) and a free N-terminal methionine (M1), each of which can form a polymer chain. Different linkage modes determine different fates: K48-linked chains mark proteins as targets for proteasomal degradation, thereby terminating signal transduction; K63-linked chains act as non-degradable scaffolds, recruiting downstream kinases to activate the NF-κB and MAPK pathways (6). This scaffold function represents a specialized form of ubiquitination because it does not eliminate the substrate but rather transduces signals—a property that distinguishes K63 chains from the canonical K48-linked degradation signal; M1 (linear) chains assembled by the LUBAC complex also promote NF-κB activation; K27-linked chains participate in immune regulation (7). Notably, compared to other cell types, macrophages express a unique set of E3 ligases and deubiquitinating enzymes (DUBs) (8). In dendritic cells, ubiquitination mainly regulates antigen presentation and co-stimulatory molecule stability (7), whereas in non-immune cells (e.g., epithelial cells, fibroblasts), the ubiquitin system primarily maintains cellular homeostasis and antiviral defenses (7). By contrast, macrophages utilize a specialized ubiquitination enzyme network—exemplified by Parkin (9) and RNF213 (10)—to integrate inflammatory tuning, pathogen targeting, and xenophagic degradation in a bacteria-specific manner. These cell-specific differences underscore the unique and indispensable role of the macrophage ubiquitination system in host antibacterial immunity.

Macrophages recognize bacteria through pattern recognition receptors (PRRs) like TLRs, subsequently activating downstream signaling pathways and producing pro-inflammatory cytokines. However, excessive inflammatory responses may cause host tissue damage, making the ubiquitin system crucial for “precision braking” of inflammatory signals (7). In the canonical TLR/NF-κB pathway, TRAF6 serves as a pivotal E3 ubiquitin ligase. It catalyzes the formation of K63-linked ubiquitin chains and couples them to downstream substrate proteins, thereby promoting NF-κB activation (11). This ubiquitin chain functions not as a signal for protein degradation, but as a molecular scaffold that recruits and activates downstream kinases. In contrast, the classical deubiquitinating enzyme A20 (TNFAIP3) functions as a major negative regulator. It inhibits TRAF6 activity by removing K63-linked ubiquitin chains from TRAF6 while simultaneously catalyzing its own K48-linked ubiquitination, promoting its degradation. This dual mechanism precisely regulates the intensity and duration of inflammatory signaling (12).

In recent years, studies have identified novel ubiquitin ligases involved in inflammation regulation, further enriching this regulatory network. TRIM21 is a unique E3 ubiquitin ligase that recognizes antibody-pathogen complexes in the cytoplasm, catalyzes their ubiquitination, and thereby promotes complement activation and inflammatory responses. This mechanism reveals TRIM21’s “gatekeeper” role in antibacterial immunity (13). Concurrently, the deubiquitinating enzyme USP7 stabilizes multiple immune-related proteins. By removing ubiquitin modifications from these proteins, it maintains the homeostasis of inflammatory signaling, preventing premature inactivation and ensuring efficient pathogen clearance (14). Certain bacteria, such as *Mtb* and *Salmonella*, can survive and replicate within macrophages. Ubiquitination plays an indispensable role in recognizing, labeling these intracellular bacteria, and directing them toward autophagosomes for degradation—a process termed xenophagy. Within the classical autophagy pathway, Parkin (PRKN) serves as a key E3 ubiquitin ligase. Its unique mechanism enables activation following bacterial invasion. Upon recruitment to bacterial surfaces, Parkin catalyzes ubiquitination. These ubiquitin chains serve as binding sites for autophagy receptors (e.g., p62/SQSTM1), effectively “pinning” bacteria to autophagosomes for eventual lysosomal degradation (9). Newly identified regulatory enzymes further expand the autophagy ubiquitination network (15). RNF213, an E3 ubiquitin ligase initially associated with vascular diseases, has drawn significant attention for its role in infection immunity. Studies reveal it recognizes and ubiquitinates invading bacteria, promoting their clearance via heterophagocytosis—offering a novel perspective linking angiogenesis to immune defense (16). Furthermore, RNF168, an E3 ubiquitin ligase crucial for DNA damage repair, was unexpectedly found to be recruited to bacterial surfaces where it catalyzes ubiquitination, thereby promoting autophagic clearance. These discoveries reveal that hosts may share partial ubiquitin-mediated regulatory pathways when confronting different threats (17).

This review systematically outlines the central role of the host ubiquitination system in macrophage immunity against bacterial infection. Through a complex “enzyme-chain-substrate” network, this system performs dual functions of maintaining dynamic equilibrium and precise regulation in two critical processes: inflammatory signaling and intracellular pathogen clearance. Deepening our understanding of the role of host ubiquitination in infection immunity not only illuminates the molecular mechanisms of host-pathogen interactions but also offers novel insights and potential therapeutic targets for combating the growing challenge of drug-resistant bacterial infections.

The following mini-glossary defines key ubiquitin chain types and related terms used in this review.

## 2 Core regulatory systems of ubiquitination in host macrophages

### 2.1 Key ubiquitinating enzymes and deubiquitinating enzymes

Macrophages constitute the core defense line of the innate immune system, playing a crucial role in pathogen clearance and maintaining bodily homeostasis. Upon bacterial invasion, macrophages rapidly recognize and phagocytose pathogens via pattern recognition receptors (PRRs), subsequently initiating defense mechanisms through a sophisticated molecular regulatory network. Within this complex and efficient defense system, ubiquitin ligases and deubiquitinating enzymes function as a pair of dynamic molecular switches. They precisely coordinate the initiation and termination of immune signaling, ensuring the body effectively and appropriately eliminates pathogens while avoiding damage to its own tissues (7). Ubiquitin ligases (E3 ligases) serve as the core executors of the ubiquitination reaction. Through a three-enzyme cascade system (E1-E2-E3), they specifically attach activated ubiquitin molecules to target proteins. The E1 activator first activates ubiquitin, which is then transferred to the E2 conjugase. Finally, the E3 ubiquitin ligase recognizes and recruits specific substrates to complete ubiquitin transfer (18). Depending on the linkage pattern between ubiquitin molecules, the resulting ubiquitin chains exhibit distinct topological structures, mediating diverse cellular fates. For example, K48-linked ubiquitin chains serve as a classic “degradation tag,” guiding ubiquitinated substrate proteins to recognize the entry structure of the proteasome and subsequently enter its core lumen for degradation. In contrast, K63-linked ubiquitin chains do not lead to degradation but function as molecular scaffolds to activate downstream signaling pathways (19).

In macrophages, TRAF6 is a key E3 ubiquitin ligase. Upon activation in the TLR4 signaling pathway, it catalyzes the formation of K63-linked ubiquitin chains via its RING-H2 domain, thereby activating the downstream kinase TAK1 and initiating NF-κB and MAPK signaling pathways. The molecular mechanism of this process involves the catalytic action of its cysteine residues, precisely transferring ubiquitin molecules from E2 to target proteins (20).Furthermore, Parkin directly targets intracellular bacteria such as *Salmonella* or Mycobacterium by ubiquitinating bacterial surfaces or the surrounding membrane, marking them for xenophagic clearance—a vital defense mechanism in macrophages (9). In summary, ubiquitin ligases provide multi-layered “initiation” and “amplification” signals for macrophage antibacterial defense through their diverse substrate specificities and types of ubiquitin chain attachment.

Corresponding to ubiquitin ligases initiating immune responses, deubiquitinating enzymes (DUBs) also play indispensable roles in immune regulation (21). These enzymes precisely cleave ubiquitin chains through their hydrolase activity, removing ubiquitin molecules from target proteins. This enables precise regulation of substrate proteins’ ubiquitinated states, maintaining cellular homeostasis and preventing excessive immune responses (22). The precision of DUBs stems from their diverse domains. For instance, members of the USP family typically contain a highly conserved catalytic triad (Cys-His-Asp/Asn), enabling them to specifically recognize and cleave ubiquitin chains (23). A20 is one of the most extensively studied DUBs, exerting negative feedback regulation during the late stages of inflammatory responses (24). Upon activation of inflammatory signals, A20 expression is induced. Its deubiquitinating activity removes K63-linked ubiquitin chains from TRAF6, thereby terminating NF-κB signaling (25). Notably, A20 itself undergoes complex regulation; its phosphorylation or ubiquitination modifications can influence enzymatic activity and subcellular localization (26–28).Another crucial DUB is USP25, which negatively regulates TLR signaling by deubiquitinating the adaptor protein TRAF3 to inhibit its degradation; its functional loss may contribute to excessive inflammation and exacerbate LPS-induced septic shock (29, 30). Indeed, dysregulation of DUB function is closely linked to various chronic inflammatory conditions, including autoimmune diseases and inflammatory bowel disease. This is because DUB imbalance can disrupt immune responses, triggering persistent pathological inflammation (31).

The synergistic interaction between ubiquitinating and deubiquitinating enzymes constitutes a dynamic and intricate process, with each playing distinct roles at different stages of macrophage antibacterial defense (21). During the early phase of bacterial invasion, ubiquitinating enzymes such as TRAF6 and LUBAC are rapidly activated. They generate K63-linked and linear ubiquitin chains, thereby swiftly initiating the NF-κB signaling pathway to trigger an inflammatory response (29, 32). As inflammation progresses and bacteria are cleared, deubiquitinating enzymes gradually increase their expression levels. They begin removing ubiquitin chains from molecules such as TRAF6 and promote their degradation, thereby “shutting down” inflammatory signaling and restoring macrophages to a quiescent state (21). This temporally coordinated, mutually restraining regulatory mechanism ensures the precision of the immune response, safeguarding both the effective defensive function of macrophages and the host’s tissue health.

### 2.2 Two core pathways of host ubiquitination

#### 2.2.1 Host ubiquitination - TLR/NF-κB pathway

In macrophages, the TLR/NF-κB pathway serves as a central axis for detecting bacterial invasion and mounting a pro-inflammatory response. Upon recognition of pathogen-associated molecular patterns (PAMPs)—such as lipoarabinomannan (LAM) from *Mtb*, peptidoglycan from *(S. aureus)*, or flagellin from *Salmonella*—by surface or endosomal Toll-like receptors (TLR4, TLR2, TLR5), a conserved intracellular signaling cascade is initiated. The adaptor protein MyD88 is recruited to the cytoplasmic domain of the TLR, which in turn recruits the E3 ubiquitin ligase TRAF6. As an E3 ubiquitin ligase, TRAF6 catalyzes the K63-linked polyubiquitination of its substrates. These K63 chains do not serve to mark proteins for degradation but rather act as a molecular scaffold to recruit and activate the kinase TAK1. Subsequently, activated TAK1 phosphorylates the IKK complex (IKKα/IKKβ/NEMO), leading to IKKβ-mediated phosphorylation of IκBα. Phosphorylated IκBα is then recognized by the SCFβ-TrCP E3 ubiquitin ligase complex, which catalyzes its K48-linked ubiquitination. The K48-linked ubiquitination tag leads to the proteasomal degradation of IκBα, thereby releasing the NF-κB dimer (p65/p50) and facilitating its transport to the cell nucleus. Once in the nucleus, NF-κB drives the transcription of pro-inflammatory cytokines such as IL-6 and TNF-α, thereby coordinating the host’s antimicrobial inflammatory response (33, 34).

While this pathway is essential for eliminating bacteria, excessive or prolonged NF-κB activation causes collateral tissue damage. The host ubiquitination system thus incorporates negative feedback mechanisms to counteract inflammation. Key among them is the deubiquitinating enzyme A20 (TNFAIP3). A20 removes K63-linked ubiquitin chains from TRAF6 via its OTU domain, thereby terminating TAK1 signaling. Additionally, A20 promotes K48-linked ubiquitination and degradation of TRAF6 (35). Another layer of control involves USP15: under steady-state conditions, USP15 deubiquitinates and stabilizes IκBα, but during inflammation, the ER-resident E3 ligase Hrd1 ubiquitinates and inactivates USP15, ensuring sustained IκBα degradation and inflammatory progression (3). Once bacterial clearance is achieved, A20 and other DUBs restore homeostasis.

**Figure 1:**
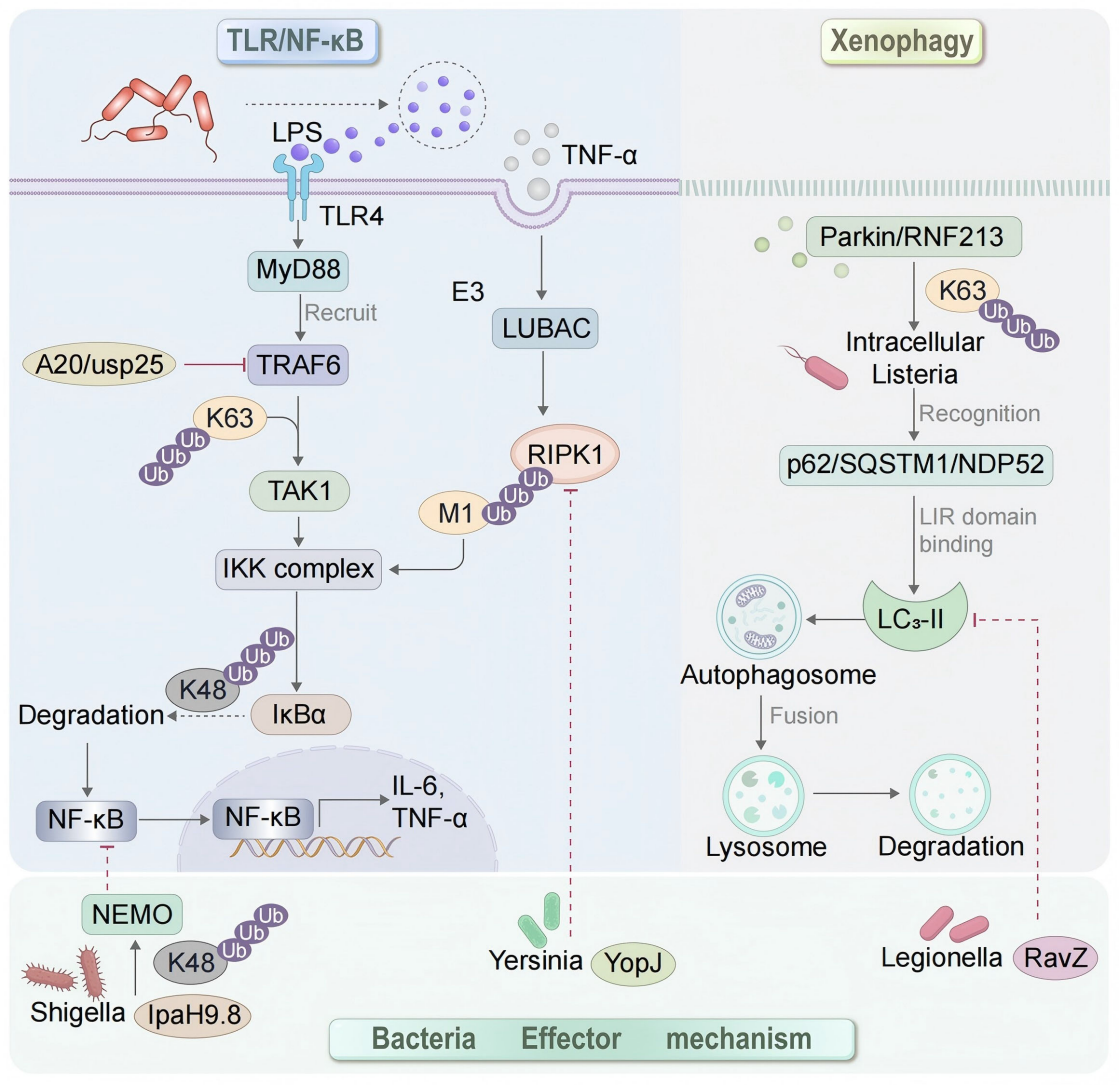
The host ubiquitination system orchestrates TLR/NF-κB signaling and xenophagy in macrophages during bacterial infection. Ubiquitin-mediated regulation of macrophage antibacterial immunity. (Left) TLR/NF-κB signaling axis. Upon LPS recognition, TLR4 recruits MyD88 and activates the E3 ubiquitin ligase TRAF6. TRAF6 catalyzes the formation of K63-linked ubiquitin chains, which serve as a scaffold to recruit and activate the TAK1 kinase complex, leading to IKK complex phosphorylation. Subsequently, IκBα undergoes K48-linked ubiquitination and proteasomal degradation, releasing NF-κB to translocate into the nucleus and drive the transcription of pro-inflammatory cytokines (e.g., IL-6, TNF-α). This process is tightly regulated by the deubiquitinating enzyme A20, which removes K63 chains from TRAF6 to prevent excessive inflammation. (Right) Xenophagy axis. Intracellular bacteria (e.g., *Salmonella*, *Mtb*) are tagged with ubiquitin chains deposited by host E3 ligases such as Parkin and RNF213. Autophagy receptors (e.g., p62/SQSTM1, NDP52) recognize these ubiquitin chains via their UBA domains and simultaneously bind LC3-II on the phagophore membrane via their LIR domains, tethering the pathogen to the forming autophagosome. Subsequent fusion with lysosomes leads to bacterial degradation. (Bottom) Bacterial countermeasures. Pathogens have evolved effector proteins to subvert ubiquitin-dependent host defenses: *Shigella* IpaH9.8 targets NEMO for K48-linked degradation; *Yersinia* YopJ inhibits kinase activation upstream of the IKK complex; *Legionella* RavZ irreversibly cleaves LC3-II to prevent autophagosome formation.

Bacteria can regulate the host TLR/NF-κB signaling pathway by secreting effectors, which can be classified into two categories based on their functional effects: overactivation and inhibition (36). One class of effectors continuously activates the NF-κB/MAPK signaling pathway through mechanisms such as enhancing K63-linked ubiquitination, directly phosphorylating IκBα, or activating Rho GTPases, thereby inducing excessive inflammatory responses. For example, *Salmonella* SopE indirectly amplifies NF-κB signaling by activating Rac1/Cdc42 (37), while *(L.pneumophila)*’s LegK1 directly phosphorylates IκBα (Ser32/36) to induce its degradation, resulting in sustained nuclear translocation of NF-κB (38). Another class of pathogens suppresses NF-κB activation by degrading key signaling molecules, blocking kinase activation, or interfering with ubiquitin chain assembly, thereby evading host immune clearance. For example, *Shigella*’s IpaH9.8 acts as an E3 ubiquitin ligase to target NEMO and catalyze its K48-ubiquitinated degradation, thereby blocking IKK complex assembly (39), while *Yersinia enterocolitica(Y. enterocolitica)* YopJ prevents the phosphorylation of MKKs, IKKβ, and RIPK1 by acetylating their activation-loop serine residues, thereby inhibiting NF-κB and MAPK signaling (40) (**Figure 1**).

In summary, the ubiquitin system’s role in macrophage anti-infective immunity extends far beyond simple protein degradation. It precisely regulates signal transduction, target protein stability, and the clearance of pathogen effector molecules through diverse ubiquitin chain types (e.g., K63, K48, K27) via the “ ubiquitination-deubiquitination balance” mechanism, collectively weaving a complex network. Understanding the intricate mechanisms of this network is crucial not only for deepening our comprehension of the dynamic processes of innate immune responses but also for providing potential directions for developing novel host-targeted therapies against pathogen escape strategies.

#### 2.2.2 Host ubiquitination-autophagy pathway

Autophagy is a conserved intracellular degradation process in which cytoplasmic components (including damaged organelles, protein aggregates, and invading pathogens) are sequestered into double-membrane vesicles called autophagosomes and delivered to lysosomes for breakdown (41). The core autophagy machinery includes: the ULK1/2 kinase complex (initiation), the class III PI3K complex containing Beclin1 (nucleation), the ATG12-ATG5-ATG16L1 conjugation system, and the LC3 lipidation system (elongation and closure) (41). In macrophages, autophagy serves as a critical innate immune mechanism against intracellular bacteria (9, 42).

Xenophagy is a specialized form of selective autophagy that targets intracellular pathogens (bacteria, viruses, parasites) (43). Unlike non-selective (starvation-induced) autophagy, selective autophagy relies on ubiquitin-binding adaptors that recognize ubiquitinated cargoes. In the context of bacterial infection, the host ubiquitination system acts as a “targeting device”: ubiquitin chains deposited on bacterial surfaces or on damaged bacteria-containing vacuoles are recognized by autophagy receptors (e.g., p62/SQSTM1, NDP52, OPTN), which simultaneously bind ubiquitin (via UBA domains) and LC3 (via LIR domains), thereby tethering the pathogen to the forming autophagosome. This “label-recognize-eliminate” sequence is the essence of xenophagy (43–45).

Mitophagy is the selective autophagic clearance of damaged or dysfunctional mitochondria. The canonical mitophagy pathway involves the PINK1/Parkin axis: loss of mitochondrial membrane potential leads to PINK1 accumulation on the outer mitochondrial membrane, which recruits and activates the E3 ubiquitin ligase Parkin. Parkin then ubiquitinates multiple outer membrane proteins, generating ubiquitin chains that serve as docking sites for autophagy receptors, culminating in mitochondrial engulfment by autophagosomes (43, 45, 46). In macrophages infected with *Salmonella* or *Mtb*, damaged mitochondria serve as the primary source of mtROS. These dysfunctional mitochondria cause electrons to leak from the mitochondrial electron transport chain (primarily at complexes I and III), reacting with oxygen to form superoxide (O_2_^-^), which is then rapidly converted by mitochondrial superoxide dismutase (SOD2) into hydrogen peroxide (H_2_O_2_). Parkin-mediated mitochondrial autophagy regulates mtROS levels by clearing these damaged mitochondria, and mtROS itself acts as a key signaling molecule involved in the host’s innate immune response against intracellular pathogens (46). During infection, bacterial components (e.g., LPS) or Parkin-mediated ubiquitination can transiently increase mtROS production. This mtROS contributes to direct oxidative killing of intracellular bacteria, synergizing with xenophagy (9, 45).

Macrophages also produce ROS independently of mitochondria, most notably via the phagocyte NADPH oxidase (NOX2, also called phox). Upon bacterial phagocytosis, NOX2 assembles on the phagosomal membrane and generates superoxide by transferring electrons from cytosolic NADPH to molecular oxygen. This process produces large amounts of ROS directly within the phagosome, independent of mitochondrial metabolism (47, 48). In contrast, mtROS production is generally lower in magnitude but can be sustained and spatially targeted to the cytosol or to damaged mitochondria. Both NOX2-derived and mtROS contribute to bacterial killing, but they are regulated differently: NOX2 is rapidly activated downstream of TLR and Fc receptor signaling, whereas mtROS elevation often accompanies mitophagy or mitochondrial dysfunction (9, 46).

When macrophages phagocytose *Mtb* or *Salmonella*, bacterial secretion systems disrupt the integrity of the vacuole, exposing the bacteria to the cytoplasm. The bacteria are tagged with ubiquitin chains and recognized by autophagy receptors, which recruit the autophagy machinery to clear the bacteria via xenophagy (49).First, ubiquitin molecules act as specialized “molecular tags,” specifically labeling invading bacteria or infected organelles, such as damaged mitochondria (42). Subsequently, autophagy receptors like p62/SQSTM1 and NDP52 act as “recognition and attachment” agents. Through their UBA domains (Ubiquitin-Associated domains), they precisely recognize and bind to ubiquitin chains on bacterial surfaces. Simultaneously, these receptors utilize their LIR domains (LC3-Interacting Region) to interact with the light chain 3 of microtubule-associated protein 1 (LC3) on the autophagosome membrane, thereby precisely recruiting ubiquitinated targets to autophagosomes (49–51). Ultimately, autophagosomes engulf the labeled bacteria and fuse with lysosomes rich in hydrolases. Within the autophagolysosomes, potent lysosomal enzymes completely degrade the bacteria, completing their clearance (52). Notably, Parkin-mediated mitochondrial ubiquitination exhibits unique bactericidal functions in this process. Upon mitochondrial damage, Parkin translocates to the outer mitochondrial membrane and catalyzes the formation of ubiquitin chains, including K63-linked polyubiquitination. K63 ubiquitination facilitates the recruitment of autophagy receptors such as p62, promoting mitochondrial sequestration and clearance via mitophagy. Concurrently, dysfunctional mitochondria generate mtROS through electron transport chain leakage. The coordinated action of Parkin-mediated ubiquitination and mtROS production synergistically enhances oxidative killing of intracellular bacteria (9, 53). The efficacy of the ubiquitination-autophagy pathway relies on a complex regulatory network, while pathogens have evolved corresponding mechanisms to evade clearance, constituting a sophisticated biological game. At the initiation stage of autophagy, Beclin1 serves as a core regulatory protein. Ubiquitin-specific protease 33 (USP33) prevents Beclin1 degradation by removing ubiquitin molecules from it, thereby maintaining its protein levels and ensuring the stability of the autophagy initiation complex (54, 55). However, pathogens like *Mtb* counter this mechanism by secreting protein kinase G (PknG). Studies have shown that PknG initiates autophagy by targeting AKT, while simultaneously inhibiting autophagosome maturation by targeting the host small GTPase RAB14 and its regulatory protein TBC1D4. This ultimately leads to a blockage in the autophagic flux, thereby creating conditions for bacterial survival within host cells (56).

In addition to PknG, other bacterial effectors target the ubiquitin-autophagy axis. *Salmonella enterica (S. enterica)* secretes SopB, a phosphoinositide phosphatase that inhibits xenophagy via dual mechanisms. SopB suppresses the terminal fusion of *Salmonella*-containing vacuoles (SCVs) with lysosomes and autophagosomes and downregulates overall lysosomal biogenesis by restricting the nuclear localization of TFEB through the Akt-TFEB axis, thereby facilitating bacterial survival in macrophages (57). *Legionella pneumophila (L. pneumophila)* employs RavZ to inhibit xenophagy. RavZ irreversibly cleaves lipidated LC3 (LC3-II) from autophagosomal membranes, preventing autophagosome maturation and bacterial capture (58). Beyond these classical pathways, recent studies have uncovered novel ubiquitin-mediated regulatory nodes that play complementary roles in host defense. The RNF213-NDP52 axis reveals an intricate mid-autophagy compensation mechanism. During *Mtb* infection, the host E3 ubiquitin ligase RNF213 is recruited to bacteria that have breached the phagosomal membrane. RNF213 directly ubiquitinates lipopolysaccharide (LPS) on the bacterial surface, generating ubiquitin chains. These ubiquitin chains are recognized by autophagy receptors including NDP52 via their UBA domains. NDP52 simultaneously binds LC3 on the autophagosome membrane through its LIR domain, thereby tethering ubiquitin-marked bacteria to the autophagosomal machinery and promoting bacterial clearance via xenophagy (10, 59) (**Figure 1**).

In summary, the ubiquitin-autophagy pathway serves as a critical “targeting system” for host clearance of intracellular bacteria. The classical “ubiquitination-autophagy receptor-autophagosome” process constitutes a fundamental immune defense, while Parkin-mediated mitochondrial autophagy enhances bactericidal effects by releasing mtROS. However, pathogens have evolved molecules like PknG to evade this defense by inhibiting autophagy initiation through Beclin1 interference. These discoveries not only deepen our understanding of host defense mechanisms but also provide crucial scientific foundations for developing more targeted novel drugs and therapeutic strategies. Future investigations into these novel mechanisms, particularly their specific functions across diverse infection models, will yield breakthroughs in effectively addressing the global challenge of intracellular bacterial infections.

## 3 E3 ligase-mediated host cell responses in different bacterial infections

### 3.1 Gram-positive bacteria

#### 3.1.1 Characteristics of gram-positive bacterial infections and the immune response of macrophages

Gram-positive bacteria lack an outer membrane but possess a thick layer of peptidoglycan, into which lipoteichoic acid (LTA) and wall teichoic acid (WTA) are embedded. These surface molecules are recognized by pattern recognition receptors (primarily TLR2 and NOD2) on macrophages, thereby triggering the production of pro-inflammatory cytokines and phagocytosis (60). Upon recognition, macrophages initiate a vigorous antimicrobial response, including the production of reactive oxygen species (ROS) and nitric oxide (NO), as well as the recruitment of autophagy mechanisms. However, these pathogens have evolved strategies to counteract macrophage-mediated killing, such as capsule formation, secretion of pore-forming toxins, interference with phagosome-lysosome fusion, and exploitation of the host’s ubiquitin system to evade immunity (2).

Notably, *Mtb* is not a typical Gram-positive bacterium due to its unique mycolic acid-rich cell wall and acid-fast staining characteristics; its cell wall does not contain LTA/WTA. Its immune recognition primarily occurs through the interaction of its unique components, such as lipoproteins and lipoarabinomannans (LAM), with the host TLR2, rather than through LTA/WTA (61). Although *Mtb* differs significantly from typical Gram-positive bacteria in its pathogenic mechanisms (such as intracellular parasitism and inhibition of phagosome maturation), both lack an outer membrane and interact with the host ubiquitin system (62). Therefore, to systematically elucidate the host ubiquitin system’s response mechanisms to major bacterial pathogens, this section will discuss typical Gram-positive bacteria and *Mtb* separately, with the aim of comprehensively understanding host defense strategies.

#### 3.1.2 *Staphylococcus aureus* and the host ubiquitin system crosstalk

*Staphylococcus aureus (S. aureus)* manipulates the host ubiquitin system through various mechanisms, while the host counteracts these efforts using deubiquitinating enzymes and microRNAs (miRNAs), among other means.

##### 3.1.2.1 Bacterial hijacking strategy

HlgB hijacks AMFR to promote excessive inflammation. The *S. aureus* virulence factor HlgB binds to the endoplasmic reticulum-resident E3 ligase AMFR, prompting AMFR to mediate K27-linked ubiquitination of TAB3, thereby activating the NF-κB/MAPK signaling pathway, inducing an excessive inflammatory response, and exacerbating lung injury (63).

EsxB targets STING to inhibit type I interferon production. Secreted via the type VII secretion system (T7SS), EsxB directly binds to the host STING protein, inhibits K63 ubiquitination of STING at the K83 site, thereby suppressing type I interferon production and helping the bacterium evade immune clearance (64).

IFP35 promotes ferroptosis by inducing Nrf2 degradation. The host protein IFP35 is upregulated during *S. aureus* infection; by interacting with Nrf2, it promotes K48 ubiquitination and proteasomal degradation of Nrf2, thereby inducing ferroptosis and exacerbating tissue damage (65).

SKP2 inhibits autophagosome formation. The E3 ligase SKP2 becomes more stable during infection and relocates to the cytoplasm, thereby inhibiting autophagosome formation. Although downregulation of SKP2 increases LC3-II levels, bacterial survival rates actually rise, suggesting that the accumulation of LC3-II may reflect an obstruction of the autophagic flux rather than enhanced autophagy (66).

In addition, while some reports have suggested that δ-hemolysin may inhibit endoplasmic reticulum-associated degradation (ERAD), direct evidence is limited, and this paper does not focus on this topic.

##### 3.1.2.2 Host defense mechanisms

The USP7-NLRP3 axis maintains a moderate inflammatory response. The deubiquitinating enzyme USP7 stabilizes NLRP3 protein levels by removing the K48 ubiquitin chain from NLRP3, ensuring moderate inflammasome activation and IL-1β release. This mechanism is particularly important when *S. aureus* suppresses NF-κB signaling (67).

USP30 antagonizes Parkin to maintain mitochondrial homeostasis. The mitochondrial deubiquitinase USP30 antagonizes Parkin-mediated mitochondrial autophagy, preventing excessive mitochondrial turnover, which may help maintain energy supply in macrophages and thereby protect the host (68).

The miR-127-A20-STAT3 axis enhances antimicrobial activity. By specifically inhibiting A20 expression, miR-127 reduces A20-mediated K63 deubiquitination of STAT3, thereby increasing STAT3 phosphorylation levels. This promotes the production of antimicrobial peptides (AMPs) and cytokines such as IL-22 and IL-17 and enhances the host’s bactericidal activity (69).

#### 3.1.3 *Streptococcus pneumoniae* and the host ubiquitin system crosstalk

*Streptococcus pneumoniae (S. pneumoniae)* is a major pathogen responsible for community-acquired pneumonia, yet research on its direct interaction with the host ubiquitin system remains relatively limited. Currently, the primary molecular evidence focuses on the regulatory role of the host E3 ligase NKLAM in anti-infectious immunity.

NKLAM positively regulates inflammatory responses and bactericidal activity. NKLAM (Natural Killer Lytic-Associated Molecule, also known as RNF19B) is an E3 ubiquitin ligase of the RBR family, expressed in macrophages and inducibly upregulated by bacterial infection. In a *S. pneumoniae* infection model, NKLAM deficiency resulted in reduced production of inflammatory cytokines and decreased bactericidal activity, suggesting that NKLAM acts as a positive regulator of host defense. Although NKLAM has been shown to mediate K63-ubiquitination of STAT1 and enhance its transcriptional activity, its specific substrates and the type of ubiquitin chain involved in *S. pneumoniae* infection remain to be further elucidated (70).

Other surface proteins, such as PspA, may evade immune clearance by inhibiting complement deposition, and the autolysin LytA may influence ISG15 modification; however, these mechanisms have a weak direct link to the classical ubiquitination pathway and require further research for validation. They are not the focus of this discussion.

#### 3.1.4 *Mycobacterium tuberculosis* and the host ubiquitin system crosstalk

*Mycobacterium tuberculosis (Mtb)* employs various strategies to manipulate the host ubiquitin system to evade the immune response, including hijacking host E3 ligases, inhibiting E3 expression, promoting substrate degradation, and binding to ubiquitin; the host, in turn, counteracts these strategies through deubiquitinating enzymes, autophagy receptors, and non-canonical pathways.

##### 3.1.4.1 Bacterial hijacking strategy

Rv0222 inhibits NF-κB through K11 ubiquitination. The *Mtb* secreted protein Rv0222 is ubiquitinated at lysine 76 via K11-linked ubiquitination by the host E3 ligase ANAPC2. Ubiquitinated Rv0222 recruits the phosphatase SHP1 to TRAF6, blocking the K63-linked ubiquitination of TRAF6, thereby inhibiting NF-κB-mediated pro-inflammatory cytokine production (71).

PPE68 suppresses inflammatory signaling by K63-ubiquitinating NF-κB. The PPE68 protein is K63-ubiquitinated at lysine 166 by the host E3 ligase MKRN1; it also suppresses the NF-κB and AP-1 signaling pathways by recruiting SHP1, thereby reducing the production of TNF-α, IL-6, and NO (72).

PtpB utilizes ubiquitin binding to activate its phosphatase activity. PtpB contains a ubiquitin-interacting motif (UIM)-like domain; upon binding to host ubiquitin, it undergoes a conformational change that activates its phospholipase activity. Activated PtpB dephosphorylates membrane phospholipids PI4P and PI (4, 5)P2, preventing the N-terminal fragment of GSDMD from localizing to the plasma membrane, thereby inhibiting pyroptosis and the release of IL-1β/IL-18 (73).

PPE36 promotes MyD88 degradation. The cell wall protein PPE36 facilitates the K48 polyubiquitination and proteasomal degradation of MyD88 mediated by the host E3 ligase Smurf1, thereby blocking the TLR signaling pathway and inhibiting the activation of NF-κB and MAPK (74).

ITCH suppression is mediated by epigenetic regulation. *Mtb* induces the expression of PRMT5 and YY1, leading to the formation of repressive histone modifications (H4R3me2) in the ITCH gene promoter region, thereby inhibiting ITCH transcription. Downregulation of ITCH prevents the degradation of its substrates ADRP and CD36, promoting lipid accumulation and the formation of foam macrophages, which create a microenvironment conducive to the long-term survival of *Mtb* (75).

LNX1 inhibition is regulated by miRNAs. *Mtb* induces the upregulation of miR-325-3p, which specifically targets and inhibits the expression of the E3 ligase LNX1, leading to the accumulation of NEK6. This, in turn, activates the anti-apoptotic STAT3 signaling pathway and inhibits macrophage apoptosis (76).

##### 3.1.4.2 Host defense mechanisms

The EST12-c-Myc-FBW7 axis enhances bactericidal activity and prevents excessive inflammation. The *Mtb*-secreted protein EST12 enters macrophages via endocytosis, binds to RACK1 to activate the JNK-AP1 signaling pathway, induces early expression of c-Myc, promotes the production of IL-6, TNF-α, and iNOS, and enhances bactericidal activity. Subsequently, the E3 ligase FBW7 catalyzes the K48-ubiquitinated degradation of c-Myc, forming a negative feedback loop that prevents excessive inflammatory damage (77).

Rv1468c triggers heterologous autophagy. The *Mtb* surface protein Rv1468c contains a UBA domain that can directly bind to host ubiquitin (without relying on an E3 ligase and without specificity for ubiquitin chain types), recruit the autophagy receptor p62 to initiate heterologous autophagy, clear some bacteria, and limit the inflammatory response (42).

Smurf1-mediated K48 ubiquitination triggers autophagy. The host E3 ligase Smurf1 targets *Mtb*-associated membrane structures via its C2 domain, catalyzes K48 polyubiquitination, and recruits the autophagy receptor NBR1 to deliver *Mtb* to autophagosomes for degradation. Smurf1 deficiency leads to increased bacterial load, exacerbated pulmonary inflammation, and higher mortality (78).

The dual regulatory roles of A20. A20 not only inhibits NF-κB signaling by deubiquitinating TRAF6 but also promotes the K48-ubiquitinated degradation of RIPK3 via the miR-342-3p/SOCS6 axis to suppress necrotic apoptosis or regulates antibacterial immunity by deubiquitinating STAT3 (69).

Non-canonical functions of TRIM27. During *Mtb* infection, TRIM27 translocates into the nucleus, where it acts as a transcriptional activator by binding to the TFEB promoter to promote the expression of autophagy-related genes; however, this function does not depend on its E3 ubiquitin ligase activity (79).

USP25 enhances bactericidal activity. The deubiquitinase USP25 stabilizes B-Raf/C-Raf through deubiquitination, activates the ERK signaling pathway, and enhances the bactericidal capacity of macrophages.

#### 3.1.5 Summary and outlook

Gram-positive bacteria have evolved various strategies to manipulate the host ubiquitin system to evade the immune response. These strategies include: hijacking the activity of host E3 ligases (e.g., Rv0222, PPE68), promoting the degradation of key signaling molecules by host E3 ligases (e.g., PPE36, the HlgB-AMFR axis), suppressing host E3 ligase expression (e.g., ITCH, LNX1), directly binding to ubiquitin to activate their own enzymatic activity (e.g., PtpB), and utilizing ubiquitin signaling to trigger selective autophagy (e.g., Rv1468c). In response to bacterial attacks, the host has similarly evolved a complex counter-regulatory network, including: deubiquitinating enzymes (e.g., USP7, USP30, A20) that maintain signaling homeostasis by removing ubiquitin chains; autophagy receptors (e.g., NBR1, p62) that recognize ubiquitinated bacteria and mediate their clearance, and miRNAs (e.g., miR-127) that indirectly enhance antimicrobial immunity by regulating the expression of deubiquitinating enzymes. Notably, the same molecule can exert opposite effects in different infection contexts (e.g., A20 both limits inflammation and may be exploited by bacteria), which profoundly illustrates the precision, dynamism, and context-dependence of ubiquitin system regulation (**Table 1**).

**Table 1:** Host E3 ubiquitin ligases and bacterial effectors involved in ubiquitin-mediated immune regulation during bacterial infections.

Bacterial Species	Related E3 ligases/effector proteins	Key Features/Mechanisms	References
*Mtb*	ANAPC2 (host)	Mediates the ubiquitination of Rv0222 at the K11 site, which in turn recruits SHP1 to inhibit TRAF6 signaling, thereby suppressing NF-κB activation capacity	(71)
	MKRN1 (host)	Mediates K63-linked ubiquitination of PPE68, which in turn recruits SHP1 to inhibit the NF-κB and AP-1 signaling pathways	(72)
	Smurf1 (host)	Targets bacterial-associated membrane structures, catalyzes K48-linked ubiquitination, and facilitates NBR1-mediated heterologous autophagy-dependent clearance of bacteria	(78)
	Parkin(host)	Mediates mitochondrial autophagy, regulates mtROS levels, and enhances bactericidal activity	(9)
	FBW7 (host)	Catalyzes the degradation of c-Myc via K48-linked ubiquitination, thereby preventing excessive inflammatory damage	(77)
*S. aureus*	AMFR(host)	Hijacked by the toxic factor HlgB, it mediates the K27-linked ubiquitination of TAB3, leading to the excessive activation of the NF-κB pathway	(63)
	SKP2 (host)	Increased stability following infection, inhibition of autophagosome formation, and disruption of the autophagic flux	(66)
	USP7 (host DUB)	Removes the K48 chain from NLRP3, stabilizes NLRP3 protein levels, and maintains a moderate inflammatory response	(67)
*S. pneumoniae*	NKLAM/RNF19B (host)	It upregulates the production of inflammatory cytokines and bactericidal activity, potentially by mediating K63-linked ubiquitination of STAT1	(70)
*Salmonella*	SopA (bacterial effector protein)	Possesses HECT-like E3 activity while simultaneously inhibiting the E3 activity of host TRIM56/TRIM65, thereby blocking K63 ubiquitination	(80)
	SseL (bacterial effector protein)	Bacterial DUB preferentially cleaves the K63 chain, thereby inhibiting recognition by the autophagy receptor p62 and autophagy-mediated clearance	(82–84)
	USP8 (host DUB)	Expression is downregulated following infection; inhibition of USP8 reduces p62 levels and limits intracellular bacterial replication	(85)
*Shigella*	IpaH9.8 (bacterial effector protein)	Targets NEMO and GBPs, catalyzing their K48-linked ubiquitination and degradation, thereby inhibiting NF-κB and GBP-mediated immune responses	(39, 86)
	IpaH1.4/IpaH2.5 (bacteria)	Targets the HOIP subunit of the LUBAC complex, catalyzes its degradation, and inhibits linear ubiquitin chain assembly and NF-κB activation	(87)
	IpaH7.8 (bacterial effector protein)	Targets GLMN, catalyzes its degradation, lifts the inhibition of cIAPs, and activates the NLRP3/NLRC4 inflammasome	(88, 89)
	IpaH4.5 (bacterial effector protein)	Directly interacts with NLRP3, regulates its stability, activates the inflammasome, and induces pyroptosis in macrophages	(90)
*L. pneumophila*	AnkB (bacterial effector protein)	Hijacks the host SCF1 E3 complex, catalyzes K48-linked ubiquitination, and promotes bacterial proliferation	(95)
	SidC/SdcA (bacterial effector proteins)	Catalyzes the ubiquitination of OTB1 at lysine residues, inhibits mTORC1 signaling, and induces autophagy	(91)
	RavD (bacterial effector protein)	Bacterial DUB that specifically cleaves M1 linear ubiquitin chains and inhibits NF-κB activation	(92)
	MavC (bacterial effector protein)	Glutaminase-like activity mediates the monoubiquitination of UBE2N, inactivating it and blocking the formation of K63 chains	(94)
	Lug14 (bacterial effector protein)	A novel E3 ligase that preferentially generates the K11 chain, targets ARIH2, and enhances NLRP3 inflammasome activation	(93)
	TRIM21 (host)	Directly targets the bacterial effector protein AnkB, mediates its K11-linked ubiquitination, and inhibits its virulence functions	(95)

Summary of key host E3 ubiquitin ligases, deubiquitinating enzymes (DUBs), and bacterial effector proteins discussed in this review. The table is organized by bacterial species and specifies the source (host or bacterium), the molecular mechanism of action, and the corresponding references. *Mtb, Mycobacterium tuberculosis; S. aureus, Staphylococcus aureus; S. pneumoniae, Streptococcus pneumoniae; L. pneumophila, Legionella pneumophila;* DUB, deubiquitinating enzyme; Ub, ubiquitin; NF-κB, nuclear factor kappa-B; GBP, guanylate-binding protein; LUBAC, linear ubiquitin chain assembly complex; NLRP3, NLR family pyrin domain containing 3; mtROS, mitochondrial reactive oxygen species.

Future research could focus on elucidating the structural basis of bacterium-host ubiquitinase interactions to develop targeted modulators, exploring the non-conventional functions of non-degradable ubiquitin chains in autophagy, clarifying the cross-talk between ubiquitin signaling and cell death, and investigating the therapeutic potential of miRNA regulation of the ubiquitin system, thereby providing new host-directed strategies for anti-infective therapy.

### 3.2 Gram-negative bacteria

#### 3.2.1 Characteristics of gram-negative bacterial infections and the immune response of macrophages

Gram-negative bacteria have a double-layered membrane structure (the outer membrane contains lipopolysaccharide, LPS) and a thinner peptidoglycan layer; some possess periplasmic spaces and Type III/IV secretion systems (T3SS/T4SS), which can directly inject effector proteins into the host cytoplasm. Common examples include *S.enterica*, *Shigella*, and *L.pneumophial*. Macrophages recognize LPS via TLR4, activating inflammatory responses and autophagy. However, effector proteins secreted by these bacteria can block NF-κB signaling, interfere with autophagosome maturation, degrade host signaling molecules, and even directly exploit the host ubiquitin system to evade the immune response.

#### 3.2.2 *Salmonella* and the host ubiquitin system crosstalk

When *Salmonella* infects macrophages, it secretes various effector proteins via the T3SS to directly manipulate the host’s ubiquitin system, while the host simultaneously activates corresponding countermeasures.

The *Salmonella* effector protein SopA has a dual function: its C-terminal catalytic domain possesses HECT-like E3 activity (the conserved C767 residue forms a thioester intermediate with ubiquitin), while simultaneously directly inhibiting the E3 activity of host TRIM56 and TRIM65 through steric hindrance, thereby blocking the K63 ubiquitination required for innate immune signaling (80). Another effector protein, SteA, targets Cullin-1, a core component of the SCF E3 complex. By interfering with the dissociation of the inhibitor Cand-1, it blocks the neddylation modification of Cullin-1, thereby inhibiting SCF complex activation. This prevents the ubiquitination and degradation of IκB, suppresses NF-κB, and ultimately inhibits the production of proinflammatory cytokines (81). Furthermore, *Salmonella* utilizes the deubiquitinating enzyme SseL, secreted via the SPI-2 T3SS, which preferentially cleaves K63 chains. By deubiquitinating ubiquitinated protein aggregates in the cytoplasm (including ALIS: ubiquitinated protein aggregates formed in response to LPS stimulation), thereby preventing the autophagy receptor p62 from recognizing and recruiting LC3, inhibiting autophagy, and promoting bacterial replication; SseL activity is critical for *Salmonella*’s ability to kill macrophages and for its virulence in mice (82–84).

Faced with such a multifaceted attack by *Salmonella*, host cells do not simply passively endure it. Research has shown that following *Salmonella* infection of macrophages, the expression of the host deubiquitinase USP8 is downregulated. Pharmacological or genetic inhibition of USP8 significantly reduces the number of intracellular *Salmonella* survivors, a mechanism linked to alterations in autophagy flux: USP8 inhibition leads to decreased expression of the autophagy receptor p62, thereby affecting autophagy flux and limiting intracellular bacterial replication. This finding suggests that USP8 acts as a negative regulator of the host’s immune response against *Salmonella*, and that the host enhances autophagy-mediated defense by downregulating USP8 (85). Thus, the interaction between *Salmonella* and the host on the ubiquitin-autophagy axis represents a dynamic process of mutual regulation.

#### 3.2.3 Shiga toxin and the host ubiquitin system crosstalk

Shiga toxin-producing *Escherichia coli(E. coli)* secretes multiple E3 ligases of the IpaH family via the T3SS, employing a “double-edged sword” strategy that simultaneously inhibits NF-κB and activates the inflammasome.

##### 3.2.3.1 Strategies for inhibiting NF-κB

IpaH9.8 targets NEMO (IKKγ) for K48-ubiquitinated degradation, thereby inhibiting NF-κB activation (39). Concurrently, IpaH9.8 also mediates the degradation of host interferon-induced guanosine-binding proteins (GBPs). GBPs (such as hGBP1 and mGBP2) are key effector molecules of the innate immune response; upon infection, they translocate to the surface of intracellular Shigella and inhibit bacterial replication. IpaH9.8 catalyzes the K48-linked polyubiquitination and proteasomal degradation of GBPs, thereby eliminating this critical antimicrobial defense. Upon infection with strains lacking IpaH9.8 or harboring mutations in the GBP-binding site, GBPs translocate to the bacterial surface and inhibit bacterial replication; conversely, GBP-deficient mice are susceptible to both wild-type and ΔipaH9.8 strains (Shigella mutants lacking the ipaH9.8 gene), confirming the central role of GBPs in anti-Shigella immunity (86). Thus, IpaH9.8 achieves a multi-tiered assault on host defense by simultaneously inhibiting the NF-κB signaling pathway and GBP-mediated innate immunity.

In addition to IpaH9.8 degrading NEMO, IpaH1.4 and IpaH2.5 target the linear ubiquitin chain assembly complex (LUBAC). These two effector proteins directly interact with the LUBAC subunit HOIL-1L, catalyzing the K48-linked polyubiquitination and proteasomal degradation of another subunit, HOIP. The degradation of HOIP leads to the inactivation of the LUBAC complex, preventing the assembly of linear ubiquitin chains. This, in turn, inhibits NF-κB nuclear translocation and pro-inflammatory cytokine production, helping Shigella evade immune surveillance (87). Unlike the Legionella effector protein RavD (which directly deubiquitinates linear chains), Shigella employs a strategy of degrading LUBAC; although their approaches differ, both ultimately inhibit NF-κB-mediated host inflammation.

##### 3.2.3.2 Strategies for activating inflammasomes

The Shigella effector protein IpaH7.8 is an E3 ubiquitin ligase that targets the host protein GLMN (glomulin) and catalyzes its ubiquitin-mediated degradation. The degradation of GLMN releases the inhibition of cIAPs, thereby activating the NLRP3 and NLRC4 inflammasomes, inducing caspase-1 activation and macrophage pyroptosis, and promoting the inflammatory response (88, 89). Additionally, Shigella utilizes another effector protein, IpaH4.5, to activate inflammasomes. IpaH4.5 directly interacts with NLRP3 and regulates NLRP3 protein stability through its E3 activity, thereby activating inflammasome signaling and inducing pyroptosis in macrophages. Shigella strains lacking IpaH4.5 exhibit significantly reduced ability to induce pyroptosis (90). Both IpaH4.5 and IpaH7.8 activate the inflammasome by targeting different molecules (NLRP3 vs. GLMN), demonstrating *Shigella*’s redundant and highly efficient pro-inflammatory strategy.

However, no clear host countermeasures against *Shigella* effector proteins have been reported in the existing literature. Although the host may be involved in regulation through E3 ligases such as TRIM21 or deubiquitinating enzymes (e.g., A20, CYLD), these mechanisms have not yet been confirmed by the literature, and the ubiquitin-mediated battle between Shigella and the host remains to be further explored.

#### 3.2.4 Legionella and the host ubiquitin system crosstalk

Legionella injects large quantities of effector proteins into host cells via the Dot/Icm (defective for organelle trafficking/intracellular multiplication) secretion system and manipulates the host ubiquitin system through various non-canonical mechanisms.

##### 3.2.4.1 Hijacking and modifying the host ubiquitination machinery

AnkB is an F-box protein capable of hijacking the host SCF1 (CRL1) E3 ligase to catalyze K48 ubiquitination on the surface of the LCV (Legionella-containing vacuole), thereby degrading host substrates and promoting bacterial proliferation.

SidC and SdcA (both E3 ligases) catalyze the lysine ubiquitination of the host DUB OTUB1 (OTU deubiquitinase, ubiquitin aldehyde binding 1), promoting the binding of OTUB1 to DEPTOR (DEP domain-containing mTOR-interacting protein), thereby inhibiting mTORC1 (mechanistic target of rapamycin kinase complex 1) signaling and inducing autophagy; in contrast, the SidE family catalyzes serine-phosphoribosyl (PR) ubiquitination of OTUB1, blocking its DUB (deubiquitinase) activity. In addition, other effector proteins (such as Lem27, DupA, DupB, SidJ, and SdjA) can antagonize these modifications, forming a finely tuned regulatory network (91).

RavD is a cysteine-dependent DUB that specifically targets linear (M1) ubiquitin chains. Localized to LCV, it clears linear chains from the LCV surface, thereby inhibiting NF-κB activation (92).

Lug14 is a novel E3 ligase that preferentially generates K11-linked ubiquitin chains, acts in synergy with the E2 enzyme UbcH5c, and whose catalytic mechanism is independent of cysteine residues (unlike typical HECT or RING-type E3s). Lug14 targets the host RBR family E3 ligase ARIH2, leading to enhanced activation of the NLRP3 inflammasome (93). This mechanism is opposite to that of RavD (which inhibits NF-κB) and instead promotes inflammation, potentially reflecting Legionella’s complex regulation of immune responses during different stages of infection or in different host cells.

MavC is a transglutaminase that catalyzes the monoubiquitination of UBE2N via an atypical covalent linkage (linking UBE2N’s Lys92/Lys94 to ubiquitin’s Gln40), with Cys74 serving as the catalytic residue. UBE2N is a key E2 enzyme involved in the synthesis of K63-linked polyubiquitin chains; its monoubiquitination leads to UBE2N inactivation, thereby blocking K63 chain formation, inhibiting the NF-κB signaling pathway, and helping Legionella evade immune clearance during the early stages of infection (94). This mechanism is entirely distinct from the classical E1-E2-E3 cascade and represents a novel strategy by which pathogens manipulate the host ubiquitin system.

##### 3.2.4.2 Direct host countermeasures against Legionella effector proteins

Faced with *Legionella’*s vast and redundant network of effector proteins, the host has evolved direct countermeasures. Studies have shown that the host E3 ubiquitin ligase TRIM21 can directly target the *Legionella* F-box effector protein AnkB, catalyzing its K11-linked polyubiquitination. This modification does not depend on the F-box domain of AnkB nor does it affect the protein’s stability, suggesting that it may regulate AnkB’s activity or its interaction with SCF1 through non-degradative mechanisms. This is the first reported case of a bacterial effector protein being ubiquitinated via a K11 link, representing a novel host countermeasure mechanism that directly modifies and regulates bacterial virulence factors (95).

Although Legionella encodes at least 13 E3 ligases and 7 DUBs, which exhibit functional redundancy and high synergy, host TRIM21-mediated K11 ubiquitination at least partially restricts the virulence function of AnkB. Further research is needed to determine whether the host possesses additional countermeasures targeting other Legionella effector proteins.

#### 3.2.5 Summary and outlook

*Salmonella, Shigella*, and *Legionella* secrete a large number of effector proteins via the T3SS/T4SS and employ various biochemical strategies to manipulate the host ubiquitin system, including hijacking host E3 activity (e.g., AnkB hijacking CRL1), degrading key host signaling molecules (e.g., IpaH9.8 degrading NEMO and GBPs, IpaH1.4/2.5 degrading LUBAC), deubiquitination (e.g., RavD deubiquitinating linear chains, SseL deubiquitinating protein aggregates), atypical ubiquitination (e.g., transglutaminase-mediated ubiquitination by MavC and PR-ubiquitination by the SidE family), modification of host DUBs (e.g., SidC/SdcA modification of OTUB1), targeted E3 complex activation (e.g., SteA inhibition of neddylation), and direct activation of inflammasomes (e.g., IpaH7.8, IpaH4.5, Lug14). The host defends itself through corresponding countermeasures, such as TRIM21-mediated K11 ubiquitination of bacterial effector proteins (against Legionella AnkB) and enhanced autophagy flux via downregulation of USP8 (against *Salmonella*) (**Table 1**). Current understanding of host countermeasures against Shigella infection remains relatively limited and warrants further investigation.

Future efforts should focus on systematically elucidating the structural basis of interactions between bacterial effector proteins and host E3/DUB complexes, developing small-molecule inhibitors targeting bacterial E3s (such as IpaH9.8, SidEs) or host E3/DUBs, investigate the prevalence of atypical ubiquitination (e.g., PR-ubiquitination, transglutaminase-mediated) during infection, and explore the potential of broad-spectrum antimicrobial therapies utilizing the host TRIM21 or autophagy receptors.

## 4 Conclusion

The host ubiquitination system functions as both a “rheostat” for inflammatory signaling and a “targeting device” for intracellular pathogen clearance in macrophage antimicrobial immunity. By deploying distinct ubiquitin chain topologies (e.g., K63 for signal transduction, K48 for proteasomal degradation, and K27/K63 for xenophagy), this system dynamically orchestrates the initiation, intensity, and resolution of immune responses while concurrently labeling invasive bacteria for autophagic elimination.

However, the evolutionary arms race between host and pathogen has led drug-resistant bacteria to develop sophisticated counter-measures. Through secreted effector proteins, these pathogens hijack or disrupt the host ubiquitin network—mimicking E3 ligases, deploying deubiquitinases, or blocking ubiquitin chain assembly—thereby evading immune clearance and promoting their own survival. This progressive adaptation has steadily eroded the efficacy of conventional antibiotics, shifting the central challenge toward understanding the molecular grammar of this host–pathogen ubiquitin dialog.

To meet this challenge, future research should prioritize three integrated directions:

1. Mechanistic precision. Decipher the spatiotemporal interplay among E3 ligases (e.g., TRAF6, Parkin, RNF213) and deubiquitinases (e.g., A20, USP25) within macrophages during bacterial infection. Particular attention should be paid to non-degradative ubiquitin signals and the structural basis of bacterial effector-host E3/DUB interactions.
2. Translational innovation. Develop selective pharmacological modulators (inhibitors or activators) targeting nodal points of the ubiquitin system, such as TRAF6-mediated K63 ubiquitination or Parkin-dependent mitophagy. Addressing specificity and delivery challenges will be key to translating these modulators into host-directed therapies.
3. Systems integration. Unify ubiquitin signaling with other immune regulatory networks (e.g., phosphorylation, acetylation, and metabolism) to construct a holistic model of host defense. Emerging technologies—including ubiquitin-omics, single-cell imaging, and AI-driven prediction of ubiquitination events—offer powerful tools to accelerate this integration.

Achieving these goals will transform our mechanistic understanding of ubiquitin biology into next-generation therapeutic strategies capable of countering the global threat of drug-resistant bacterial infections.

## Mini Glossary

E3 ligase: An enzyme that attaches ubiquitin to a specific target protein, determining the specificity of ubiquitination.
DUB (deubiquitinating enzyme): An enzyme that removes ubiquitin chains from proteins, reversing the action of E3 ligases and terminating signals.
K48-linked ubiquitin chains: Ubiquitin chains linked via lysine 48; they target proteins for degradation by the proteasome.
K63-linked ubiquitin chains: Ubiquitin chains linked via lysine 63; they act as scaffolds to activate signaling pathways such as NF-κB.
K27-linked ubiquitin chains: A less common ubiquitin linkage type (via lysine 27) that has been implicated in regulating protein function and innate immune signaling.
K11-linked ubiquitin chains: Ubiquitin chains linked via lysine 11; involved in cell cycle control and immune regulation.
M1 (linear) ubiquitin chains: Ubiquitin chains linked via the N-terminal methionine; assembled by the LUBAC complex, they promote NF-κB activation.
Xenophagy: A selective form of autophagy that eliminates intracellular pathogens (e.g., bacteria) by engulfing them into autophagosomes for lysosomal degradation.
